# The relationship between changes in serum myostatin and adiponectin levels in patients with obesity undergoing a weight loss program

**DOI:** 10.1186/s12902-021-00808-4

**Published:** 2021-07-08

**Authors:** Nana Takao, Satoshi Kurose, Takumi Miyauchi, Katsuko Onishi, Atsuko Tamanoi, Ryota Tsuyuguchi, Aya Fujii, Sawako Yoshiuchi, Kazuhisa Takahashi, Hiromi Tsutsumi, Yutaka Kimura

**Affiliations:** 1 Department of Health Science, Graduate School of Medicine, Kansai Medical University, 2-5-1, Shinmachi, Hirakata, 573–1010 Japan; 2 Health Science Center, Kansai Medical University Hospital, 2-3-1, Shinmachi, Hirakata, 573–1191 Japan; 3 Department of Health Science, Kansai Medical University, 2-5-1, Shinmachi, Hirakata, 573–1010 Japan; 4 Department of Nutrition Management, Kansai Medical University Hospital, 2-3-1, Shinmachi, Hirakata, 573–1191 Japan; 5 Department of Medicine II, Kansai Medical University, 2-5-1 Shinmachi, Hirakata, Osaka, Japan

**Keywords:** Body composition, Lean mass, Myostatin, Adiponectin, Weight loss

## Abstract

**Background:**

An effective strategy for weight loss in patients who are overweight or obese is to reduce body fat mass while maintaining skeletal muscle mass. Adiponectin and myostatin are affected through changes in body composition due to weight loss, and examining their dynamics may contribute to strategies for maintaining skeletal muscle mass through weight loss. We aimed to examine the relationships among myostatin, adiponectin, and body composition, depending on the extent of weight loss, in patients with obesity undergoing a weight loss program.

**Methods:**

We examined 66 patients with obesity (age: 46.8 ± 14.0 years, body mass index: 34.3 [31.0–38.4] kg/m^2^) attending a hospital weight loss program. We categorized the patients into two groups, namely an L group (those with a weight reduction of < 5% from baseline) and an M group (those with a weight reduction of > 5% from baseline). All patients underwent blood tests and were assessed for body composition, insulin resistance, adipocytokine and myokine levels, exercise tolerance, and muscle strength at baseline and post-intervention.

**Results:**

Serum myostatin and adiponectin levels increased post-intervention in both groups. Body weight and %fat decreased, and the rate of lean body mass (%LBM) increased in both groups. Exercise capacity and muscle strength improved in the M group only. Change in (⊿) myostatin correlated with ⊿%fat, ⊿%LBM, and ⊿adiponectin. ⊿adiponectin (β = − 0.262, *p* = 0.035) was an independent predictor of ⊿myostatin.

**Conclusions:**

Myostatin and adiponectin might cross-talk and regulate changes in skeletal muscle and fat mass with or without successful weight loss. These findings indicate that evaluating serum myostatin and adiponectin levels in clinical practice could be used to predict the effects of weight loss and help prevent skeletal muscle mass loss.

## Background

Overweight and obesity are major risk factors for chronic diseases such as diabetes mellitus (DM), cardiovascular diseases, and cancer as well as a decreased quality of life [1]. The optimal weight loss method for people who are overweight or obese is to reduce body fat mass while maintaining skeletal muscle mass. However, skeletal muscle mass often decreases with weight loss; therefore, identifying markers that facilitate the early detection of skeletal muscle mass loss should contribute to the development of optimal treatments for obesity.

Previously, we reported that adiponectin was negatively correlated with muscle strength and that myostatin was positively correlated with appendicular lean mass [2]. Recent studies have found that skeletal muscle secretes adiponectin, which then acts as a signal to maintain skeletal muscle function and works as a metabolic regulator [3, 4]. It has been reported that higher blood adiponectin levels are associated with lower muscle mass, lower limb muscle strength, and lower handgrip strength in older adults [5, 6]. These studies showed that adiponectin acts as a conventional beneficial adipokine and has a relationship with skeletal muscle dysfunction. Myostatin, a member of the TGF-β superfamily, is a skeletal muscle-secreted myokine protein that acts in the inhibitory system of skeletal muscle formation [7]. Myostatin inhibition contributes to reducing fat accumulation through increasing muscle mass and strength [8].

Some studies have shown a relationship between adiponectin or myostatin and weight loss, diet intervention, or exercise [9–14]. Diet-induced weight and body fat loss have been associated with increased adiponectin concentrations in adults with obesity [10, 13]. Additionally, a four-week hypocaloric diet and physical exercise program in people with severe obesity was shown to result in metabolic amelioration associated with a significant increase in adiponectin levels [14]. In terms of the relationship between weight loss, exercise programs, and myokines, middle-aged men who undertook 6 months of aerobic exercise were found to have decreased myostatin levels, despite no changes in body weight [9]. Furthermore, an aerobic exercise training program combined with modest weight loss in adults with obesity was shown to have significantly reduced myostatin mRNA expression with improved insulin sensitivity [11]. Given these results, it is possible that adiponectin and myostatin cross-talk to regulate skeletal muscle and adipose tissue; however, no observational studies have been undertaken in humans.

Generally, a clinically meaningful weight loss for individuals with obesity is considered to involve a weight loss of ≥5% of the initial body weight. Such a weight loss has been associated with moderate improvements in blood pressure, low-density lipoprotein cholesterol, triglycerides, and glucose levels [15–17]. Adiponectin and myostatin are affected through changes in body composition due to weight loss, and examining their dynamics may contribute to strategies for maintaining skeletal muscle mass through weight loss. In addition, changes in adiponectin and myostatin levels may be used as early markers of skeletal muscle mass loss.

Therefore, this retrospective study aimed to examine changes in adiponectin and myostatin levels with and without successful weight loss and to investigate the association between changes in body composition and determinants of change in myostatin levels.

## Methods

### Patients

Our study comprised 66 patients with obesity (body mass index [BMI] > 30) who attended an obesity treatment program (intervention) at the Health Science Center, Kansai Medical University Hospital, Japan, between October 2014 and October 2018. Exclusion criteria comprised: patients with a BMI > 60; patients who were pregnant; patients with severe liver dysfunction, renal disease, or secondary causes of obesity due to endocrine disorders; and those with a debilitating disease.

Patients were categorized into two groups: an L group (those with a weight loss of < 5% of the baseline value) and an M group (those with a weight loss of > 5% of the baseline value) [15–17].

Body composition, blood sampling, exercise tolerance, and muscle strength (handgrip and lower limb muscle strength) were measured in all patients, and the patients completed an international physical activity questionnaire at baseline and after completion of a 6-month weight loss program. Medical history and clinical characteristics were collected from the patients’ medical records.

This study was approved by the Ethics Committee of Kansai Medical University (approval no. 2019092). All procedures performed in the study involving human participants were performed in accordance with the 1964 Helsinki Declaration and its later amendments. Written informed consent was obtained from all participants prior to commencement of the study.

### Obesity treatment program

The obesity treatment program consisted of exercise, nutritional, and psychological counseling [18, 19]. Our intervention, undertaken at the hospital, comprised 30 min of aerobic exercise in which the intensity was adjusted to the anaerobic threshold, resistance training, and stretching. Patients also performed exercise three times per week, including home-based exercise. A health fitness programmer provided supervised individual exercise program based on the exercise capacity of patients at each visit. They also provided home exercise menu for each patients. A dietician provided monthly nutritional guidance, education concerning eating behavior, and dietary instruction once a month, based on dietary record data. Using the patients’ weight records, a clinical psychologist provided psychological counseling once a month, based on cognitive behavioral therapy, and focused on self-monitoring and self-efficacy.

### Measurement of body composition

Body composition was measured using dual-energy X-ray absorptiometry (DXA, DPX-NT System, GE Healthcare, Buckinghamshire, UK). The measurement parameters included weight, fat mass, and lean body mass (LBM) (whole body, upper extremities, body trunk, and lower extremities). The rates of fat mass (%fat) and LBM (%LBM) were calculated as fat mass and LBM divided by body weight, respectively. Visceral fat area (VFA) and subcutaneous fat area (SFA) at the umbilical level were measured using computed tomography and fat scan analysis software (East Japan Technology Tokyo Laboratory, Tokyo, Japan), respectively.

### Blood sampling and measurement of serum adipokine and myokine levels

Patient medical history and clinical characteristics were collected from medical records. Fasting blood was analyzed to determine glucose (GLU), glycosylated hemoglobin (HbA1c), and immunoreactive insulin (IRI) levels. We evaluated the endogenous effect of insulin resistance on vascular function. Insulin resistance was assessed using the homeostasis model assessment of insulin resistance (HOMA-IR). HOMA-IR was calculated as follows: HOMA-IR = (IRI × fasting plasma GLU) / 405. Additionally, we measured the plasma levels of myokines, adiponectin, leptin, and irisin. Blood samples were stored at − 80 °C, and both myokine and adipokine levels were measured according to the manufacturer’s instructions. Serum myostatin and irisin levels as myokines were measured using the GDF-8/Myostatin Quantikine ELISA Kit (R&D Systems, Minneapolis, MN, USA) and human EIA Kit (Phoenix Pharmaceuticals Inc., Burlingame, CA, USA). Serum adiponectin and leptin levels as adipokines were measured using the human Quantikine ELISA Kit (R&D Systems, respectively. Minneapolis, MN, USA). The intra- and inter-assay coefficients of variation were 2.5–4.7% and 5.8–6.9% for adiponectin, 3.0–3.3% and 3.5–5.4% for leptin, 1.8–5.4% and 3.6–6.0% for myostatin, and < 10% and < 15% for irisin, respectively.

### Cardiopulmonary exercise test

A symptom-limited exercise stress test, using the ramp method, was conducted using an expiration gas analyzer (AE300S, Minato Medical Science Co., Ltd., Osaka, Japan) and an ergometer cycle (AEROBIKE 75XL, Combi, Tokyo, Japan) with a 12-lead electrocardiogram. Exercise began with a four-minute warm-up at 10–20 W and 50 rpm, followed by the 10–20 W ramp method after a five-minute rest on the ergometer. Heart rate, oxygen uptake (VO_2_), and carbon dioxide excretion volume (VCO_2_) were measured at the point of rest, warm-up, anaerobic threshold (AT), and maximum oxygen uptake (peak VO_2_) using the breath-by-breath method. The AT was determined using the V-slope method. Peak VO_2_ and work rate were defined as the peak values during incremental exercise [20].

### Muscle strength

Muscle strength and performance were measured using handgrip strength and lower limb muscle strength. Handgrip strength was measured using a handgrip dynamometer (T.K.K.5401, Takei Scientific Instruments, Niigata, Japan). Right- and left-hand grip strength was measured three times on each side, and the mean measurement was recorded. Lower limb muscle strength was measured twice for isokinetic output torque using a recumbent ergometer (Strength Ergo, Mitsubishi Electric Corp., Tokyo, Japan). We recorded the maximum value and divided the values by body weight (N∙m/kg).

### International physical activity questionnaire

Physical activity was self-reported using a shortened version of the international physical activity questionnaire [21]. The questionnaire comprised seven questions assessing physical activity in the past week. Metabolic equivalent (MET) values for walking, average physical activity, and intense physical activity were computed as 3.3, 4, and 8, respectively. The total amount of physical activity per week (day × minute × MET) was calculated through aggregating the amount of walking, moderate physical activity, and intense physical activity.

### Statistical analysis

The measured values were expressed as mean ± standard deviation or median (25, 75% quartile), and categorical data were expressed as incidences and percentages. Changes (⊿) were calculated as differences between pre- and post-intervention. Normal distribution was confirmed using the Shapiro-Wilk test. An unpaired *t*-test, a Mann-Whitney *U* test, or a Chi-squared test was used for inter-group comparisons. A paired *t*-test and a Wilcoxon signed-rank test were used for pre- and post-intervention comparisons, respectively. Correlations between myostatin and other parameters were determined using Pearson’s or Spearman’s rank correlation coefficient. A stepwise multiple regression analysis was used for multivariable analysis to examine independent predictors of ⊿myostatin. The parameters of significant correlation with ⊿myostatin were used as independent variables, and sex, age, and DM morbidity were used as adjustment factors. A *p*-value < 0.05 was considered statistically significant. All statistical analyses were conducted using SPSS version 23.0 for Windows (IBM Corp., Armonk, NY, USA) software.

## Results

### Patient baseline characteristics and parameter changes post-intervention

Patient characteristics and changes in parameters are presented in Table 1. The mean patient age was 46.8 ± 14.0, 27.3% of patients were male, the mean BMI was 34.3 kg/m^2^ (25% quartile, 31.0 kg/m^2^; 75% quartile, 38.4 kg/m^2^), and 24.2% of the patients had a diagnosis of DM.

**Table 1:** Participants’ clinical characteristics and parameter changes pre- and post-intervention

		Pre-intervention	Post-intervention	***p***-value
Age	years	46.8 ± 14.0		
Sex	M/F (%)	18 (27.3)/48 (72.7)		
Period	days	199.8 ± 23.1		
Coronary risk factors				
DM	n (%)	16 (24.2)		
Hypertension	n (%)	30 (45.5)		
Dyslipidemia	n (%)	19 (28.8)		
Alcohol consumption	n (%)	29 (43.9)		
Current smoker	n (%)	5 (7.6)		
Exercise habits	n (%)	21 (31.8)		
Body composition				
Weight	kg	90.2 (78.5–102.3)	85.1 (73.8–96.4)	< 0.001
BMI	kg/m^2^	34.3 (31.0–38.4)	32.1 (29.0–35.9)	< 0.001
%fat	%	45.3 (40.8–49.8)	43.6 (38.5–49.5)	< 0.001
%LBM	%	52.9 (48.4–57.5)	55.1 (49.0–59.5)	< 0.001
VFA	cm^3^	183.0 ± 52.7	158.3 ± 63.5	< 0.001
SFA	cm^3^	419.5 ± 161.5	374.9 ± 160.3	< 0.001
Exercise capacity and muscle strength				
ATVO_2_	ml/kg/min	11.2 (9.8–12.5)	11.7 (10.4–13.1)	0.002
Peak VO_2_	ml/kg/min	18.0 (15.5–20.3)	19.6 (16.2–23.2)	< 0.001
Handgrip	kg	26.4 (20.2–30.5)	26.2 (20.9–32.0)	0.214
Leg strength	N∙m/kg	1.47 ± 0.32	1.53 ± 0.39	< 0.001
Physical activity	kcal/day	55.1 (21.4–151.1)	173.9 (90.0–300.6)	0.004
Blood sample				
Fasting GLU	mg/dL	97 (89.8–106.3)	95 (88.0–100.5)	0.024
HbA1c	%	5.8 (5.6–6.3)	5.6 (5.4–6.0)	< 0.001
Fasting insulin	μU/mL	14.6 (9.2–19.4)	12.1 (8.5–21.9)	0.904
HOMA-IR		3.4 (2.1–4.8)	2.9 (1.9–5.2)	0.546
Myostatin	μg/mL	2377.5 (1759.9–2926.3)	2502.3 (2209.7–3548.7)	< 0.001
Adiponectin	pg/mL	2.87 (1.68–4.24)	4.22 (2.98–6.05)	< 0.001
Leptin	ng/mL	22.7 (15.4–36.3)	14.5 (8.0–23.9)	< 0.001
Irisin	ng/mL	23.2 (19.5–27.7)	17.6 (16.7–18.7)	< 0.001

Results are expressed as mean ± standard deviation or median (25, 75% quartile)

%fat, fat mass/weight × 100

%LBM, lean body mass/weight × 100

*AT* anaerobic threshold, *BMI* body mass index, *DM* diabetes mellitus, *F* female, *GLU* glucose, *HbA1c* hemoglobin Alc, *HOMA-IR* homeostasis model assessment-insulin resistance, *IRI* immunoreactive insulin, *M* male, *SFA* subcutaneous fat area, *VFA* visceral fat area, *VO*_*2*_ oxygen consumption

BMI (from 34.3 kg/m^2^ [range, 31.0–38.4] to 32.1 kg/m^2^ [range, 29.0–35.9]), %fat (from 45.3% [range, 40.8–49.8] to 43.6% [range, 38.5–49.5]), and %LBM (from 52.9% [range, 48.4–57.5] to 55.1% [range, 49.0–59.5]) significantly decreased post-intervention. VFA, SFA, exercise capacity, leg strength, physical activity, fasting GLU, and HbA1c also significantly improved post-intervention. Serum myostatin (2377.5 [range, 1759.9–2926.3] to 2502.3 [range, 2209.7–3548.7] pg/mL) and adiponectin (2.87 [range, 1.68–4.24] to 4.22 [range, 2.98–6.05] μg/mL) levels significantly increased, and leptin and irisin levels significantly decreased after the program.

**Table 2:** Parameter changes from baseline to after weight reduction intervention in the two groups

		L group (***n*** = 22)		***p***-value	M group (***n*** = 44)		***p***-value
		Pre-intervention	Post-intervention		Pre-intervention	Post-intervention	
Period	days	204.1 ± 18.6			197.6 ± 24.9		
Age	years	49.1 ± 14.3			45.7 ± 14.0		
Sex	M/F (%)	5 (22.7)/17 (77.3)			14 (31.8)/30 (68.2)		
Diabetes mellitus	n (%)	9 (40.9)			7 (15.9)*		
Hypertension	n (%)	15 (68.2)			15 (34.1)**		
Dyslipidemia	n (%)	5 (22.7)			14 (31.8)		
Body composition							
Weight	kg	86.7 (77.9–103.4)	84.9 (75.6–104.2)	0.001	95.6 ± 15.3	84.4 ± 14.6	< 0.001
BMI	kg/m^2^	35.6 ± 6.7	34.9 ± 6.9	0.002	34.5 (31.2–38.4)	31.7 (28.3–34.8)	< 0.001
%fat	%	44.8 ± 6.5	43.9 ± 6.5	0.010	44.9 ± 6.5	41.9 ± 8.8	< 0.001
%LBM	%	53.5 ± 6.2	54.4 ± 6.1	0.006	53.2 ± 6.1	56.4 ± 8.2	< 0.001
VFA	cm^3^	186.6 ± 47.0	181.6 ± 69.5	0.211	181.3 ± 55.8	146.7 ± 57.6	< 0.001
SFA	cm^3^	422.3 ± 190.8	402 ± 178.8	0.021	418.0 ± 147.0	361.2 ± 150.5	< 0.001
Exercise capacity and muscle strength							
ATVO_2_	ml/kg/min	11.0 ± 1.6	11.3 ± 1.9	0.315	11.4 (9.8–12.5)	11.8 (10.8–13.5)	0.002
PeakVO_2_	ml/kg/min	17.4 ± 3.3	17.6 ± 3.9	0.618	18.3 (15.7–20.4)	20.5 (17.7–23.9)	< 0.001
Handgrip	Kg	26.0 ± 7.1	24.4 ± 7.9	0.293	26.5 ± 9.0	28.1 ± 7.5	0.032
Leg strength	N∙m/kg	1.48 ± 0.27	1.45 ± 0.34	0.837	1.46 ± 0.34	1.56 ± 0.41	< 0.001
Physical activity	kcal/day	99.0 (14.3–274.3)	139.3 (26.8–219.6)	0.575	53.6 (21.4–120.0)	198.0 (107.1–353.6)	0.002
Blood sample							
Fasting GLU	mg/dL	104.5 ± 12.4	102.9 ± 16.3	0.586	95.5 (87.0–102.8)**	94.0 (87.3–97.8)	0.047
HbA1c	%	6.0 (5.6–6.3)	6.0 (5.5–6.2)	0.757	5.8 (5.5–6.1)	5.6 (5.4–5.8)	< 0.001
Fasting insulin	μU/mL	14.9 (8.5–17.9)	11.4 (8.5–22.0)	0.270	14.3 (9.6–19.7)	12.6 (8.0–20.8)	0.436
HOMA-IR		3.5 (2.3–4.6)	2.9 (2.0–5.7)	0.372	3.4 (2.0–5.0)	2.9 (1.8–5.0)	0.165
Adiponectin	μg/mL	2.86 (1.81–3.81)	3.06 (1.63–4.25)	< 0.001	4.15 (3.21–5.35)	4.23 (2.86–6.22)	< 0.001
Myostatin	pg/mL	2381.1 ± 760.8	2980.0 ± 1088.9	0.003	2377.5 (1751.1–2910.3)	2472.3 (2150.9–3472.3)	0.002
Leptin	ng/mL	21.2 (15.4–38.0)	14.2 (9.1–32.6)	0.004	24.1 (15.0–35.7)	15.0 (7.4–22.0)	< 0.001
Irisin	ng/mL	24.8 (20.1–27.3)	17.5 (16.8–18.7)	< 0.001	22.8 (18.8–27.7)	17.6 (16.5–18.8)	< 0.001

Results are expressed as mean ± standard deviation or median (25, 75% quartile)

L group, < 5% of weight loss; M group, > 5% of weight loss

% of sex, DM, hypertension, and dyslipidemia are shown as percentages of group members

*p*-values show comparisons between pre- and post-intervention groups

*p < 0.05, **p < 0.01, ***p < 0.001 for comparisons between < 5% weight loss and > 5% weight loss groups pre-intervention

%fat, fat mass/weight × 100

%LBM, lean body mass/weight × 100

*AT* anaerobic threshold, *BMI* body mass index, *DM* diabetes mellitus, *F* female, *GLU* glucose, *HbA1c* hemoglobin Alc, *HOMA-IR* homeostasis model assessment-insulin resistance, *IRI* immunoreactive insulin, *M* male, *SFA* subcutaneous fat area, *VFA* visceral fat area, *VO*_*2*_ oxygen consumption

### Comparison of the baseline characteristics and parameter changes in the two groups

A comparisons of the baseline characteristics and parameter changes post-intervention in the two groups is presented in Table 2.

Intervention time, age, and sex did not significantly differ between the groups. The L group comprised a large number of patients with DM (*p* < 0.05) and hypertension (*p* < 0.01). Fasting GLU was also significantly higher in the L group (*p* < 0.01). However, there were no other significant between-group differences pre-intervention.

All the parameters of body composition, except VFA in the L group, significantly improved in both groups. Exercise capacity, muscle strength, and fasting GLU levels significantly improved in the M group. Fasting insulin and HOMA-IR levels did not improve in either of the groups. Serum myostatin, adiponectin, leptin, and irisin levels changed significantly in both groups post-intervention.

### Correlation of the changing %LBM and myostatin with other parameters and stepwise multiple regression analysis of myostatin changes

The correlation between changes in %LBM and in myostatin and adiponectin levels, and changes in the other parameters are presented in Table 3.

**Table 3:** Correlations between changing weight, %lean body mass, myostatin, and other parameters

	⊿% LBM		⊿myostatin		⊿adiponectin	
	r	p	r	p	r	p
**⊿Weight**	−0.508	< 0.001	0.056	0.655	−0.045	0.718
**⊿BMI**	−0.484	<0.001	0.075	0.547	−0.061	0.626
**⊿% fat**	−0.892	<0.001	0.347	0.004	− 0.149	0.232
**⊿% LBM**	–	–	−0.359	0.003	0.102	0.413
**⊿VFA**	−0.363	0.003	−0.012	0.925	0.041	0.742
**⊿SFA**	−0.519	<0.001	−0.101	0.421	−0.092	0.463
**⊿Peak VO**_**2**_	0.315	0.011	−0.101	0.423	0.022	0.862
**⊿Handgrip**	0.048	0.709	0.072	0.577	−0.253	0.047
**⊿HOMA**	−0.162	0.194	−0.024	0.848	0.079	0.531
**⊿Myostatin**	−0.359	0.003	–	–	−0.250	0.043
**⊿Adiponectin**	0.102	0.413	−0.250	0.043	–	–
**⊿Leptin**	0.010	0.938	−0.114	0.362	−0.087	0.487
**⊿Irisin**	0.089	0.180	−0.024	0.851	−0.134	0.282

Values are expressed as correlation coefficients

⊿ signifies change in the corresponding quantity

%fat, fat mass/weight × 100

%LBM, lean body mass/weight ×100

*BMI* body mass index, *HOMA-IR* homeostasis model assessment-insulin resistance, *SFA* subcutaneous fat area, *VFA* visceral fat area, *VO*_*2*_ oxygen consumption

**Fig. 1:**
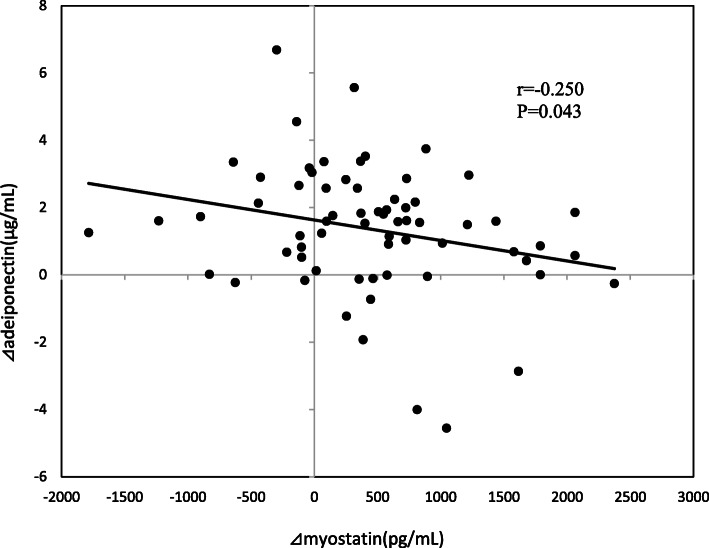
The correlation between changes in myostatin and adiponectin levels. Data were analyzed using a Spearman’s rank correlation coefficient

There were significant correlations between ⊿%LBM and ⊿weight (*r* = − 0.508, *p* < 0.001), ⊿BMI (*r* = − 0.484, *p* < 0.001), ⊿% fat (*r* = − 0.892, *p* < 0.001), ⊿VFA (*r* = − 0.363, *p* = 0.003), ⊿SFA (*r* = − 0.519, *p* < 0.001), ⊿peak VO_2_ (*r* = 0.315, *p* = 0.011), and ⊿myostatin (*r* = − 0.359, *p* = 0.003). There were significant correlations between ⊿myostatin and ⊿%fat (*r* = 0.347, *p* = 0.004), and between ⊿%LBM (*r* = − 0.359, *p* = 0.003), and ⊿adiponectin (*r* = − 0.250, *p* = 0.043; Fig. 1). ⊿adiponectin significantly correlated with ⊿handgrip (*r* = − 0.253, *p* = 0.047) and ⊿myostatin (*r* = − 0.250, *p* = 0.043).

Table 4 shows the forward-backward stepwise multivariate regression analysis for identifying independent predictors of ⊿myostatin. The independent variables examined for ⊿myostatin were sex, age, DM, ⊿%fat, ⊿%LBM, and ⊿adiponectin, based on the results of the univariate analysis. Stepwise multiple regression analysis identified ⊿adiponectin (β = − 0.262, *p* = 0.035) as an independent predictor of changing myostatin factors.

**Table 4:** Stepwise multiple regression analysis of the changes in myostatin

	β	***p***-value	VIF
Sex	0.110	0.388	1.051
Age	−0.026	0.838	1.002
DM	−0.062	0.624	1.101
⊿%fat	0.052	0.684	1.004
⊿%LBM	−0.095	0.454	1.000
⊿Adiponectin	−0.262	0.035	1.000

β: standardized partial regression coefficient

⊿ signifies change in the corresponding quantity

%fat, fat mass/weight × 100

% LBM, lean body mass/weight ×100

*DM* diabetes mellitus, *VIF* variance inflation factor

## Discussion

In our study, the mean weight loss was 7.5%, and body composition, exercise capacity, and leg strength measurements improved post-intervention in all patients. Serum myostatin and adiponectin levels increased and leptin and irisin levels decreased post-intervention. Serum myostatin and adiponectin levels significantly increased with or without 5% weight loss, but with no significant between-group differences. Changes in LBM/body weight and serum adiponectin correlated with changes in serum myostatin, and the change in the serum adiponectin level was an independent predictor of change in serum myostatin. Therefore, our study findings suggested that serum myostatin and adiponectin might control one another and affect changes in body composition regardless of successful weight loss. This is the first report to confirm the relationship between changes in serum myostatin and adiponectin levels that reflect changes in the body composition of patients with obesity following a weight loss obesity program.

Serum adiponectin levels significantly increased post-intervention in all the study patients, which is consistent with results reported in recent studies [10, 13, 14, 22]. However, although there were few reports showing decreases or no changes in serum myostatin levels following weight maintenance programs and exercise in recent studies [7, 9, 11, 12], this level did increase after weight loss in our study. We also analyzed differences in parameters between < 5% or > 5% weight loss and showed that serum myostatin levels significantly increased after weight loss in both groups, regardless of the amount of weight lost. In our previous cross-sectional study, we reported that serum myostatin positively correlated with skeletal muscle/body weight but not with absolute skeletal muscle mass [2]. We interpreted this finding to mean that myostatin levels are controlled in relation to skeletal muscle mass per body weight, to prevent overgrowth of skeletal muscle. In our longitudinal study, the change in myostatin was negatively associated with changes in skeletal muscle indicators; therefore, it is important to suppress an increase in myostatin to prevent the loss of skeletal muscle mass in a weight loss program. Therefore, we evaluated the serum myostatin level pre- and post-intervention and consider that awareness of specific changes in body composition during obesity weight loss treatment may guide optimal weight loss treatment strategies in preventing loss of skeletal muscle mass and decreasing body fat mass.

We hypothesized that changes in myostatin and adiponectin levels would affect the degree of weight loss, but we found no relationship between myostatin and adiponectin levels and weight loss. Therefore, we analyzed the relationship between myostatin and adiponectin levels and the rate of LBM divided by body weight using all the data. Changes in myostatin levels also negatively correlated with changes in adiponectin levels in our study. Additionally, we found that a change in the adiponectin level was an independent predictor of change in the myostatin level. Changes in myostatin and adiponectin levels due to weight loss might be interrelated, and myostatin might have influenced the change in LBM/body weight. Adiponectin, an important adipocytokine produced mainly by adipocytes, protects against a variety of obesity-related medical conditions. Adiponectin is inversely correlated with obesity and is tightly regulated at the transcriptional and translational levels. Myostatin has a considerable effect on the growth and development of skeletal muscle, such that genetic deletions or mutations in the myostatin gene cause a dramatic increase in skeletal muscle mass and a decrease in fat mass [23]. Adiponectin works in concert with other important hormones including insulin, leptin, and various cytokines [24], and certain myokines and adipokines interact with each other [25]. However, the extent of any cross-talk between myostatin and adiponectin has not been clarified. Some studies have reported findings concerning myostatin and adiponectin [26, 27]; recent data suggests cross-talk between myostatin-induced Smad2/3 and adiponectin-induced AMP-activated kinase/peroxisome proliferator-activated receptor α pathways [26]. Another study in mice showed that myostatin affects adipocyte formation both in vitro and in vivo [27]. Although the mechanisms involved in the relationship between myostatin and adiponectin was unclear in our study, these recent reports help to understand the mechanisms involved in our results. It is necessary to suppress myostatin secretion to prevent loss of skeletal muscle mass owing to weight loss treatment. Reducing or suppressing an increase in myostatin secretion can be expected to contribute to an increase in energy expenditure through preventing skeletal muscle atrophy, leading to the enhancement of secondary fat metabolism and changes in systemic fat and glucose metabolism. As a result, adiponectin secretion increased, and the changes in myostatin and adiponectin levels appeared to show a negative correlation in this study. One advantage of our study was that our clinical data findings indicated that myostatin and adiponectin showed potential cross-talk, which might affect lean mass in individuals with obesity undergoing a weight loss program. This finding might be informative in creating an optimal weight loss program through encouraging an evaluation of serum myostatin and adiponectin levels in clinical practice.

This study had several limitations. First, sex differences were not considered. A recent study reported elevated serum myostatin levels in male patients compared with female patients [28]. Plasma adiponectin levels in rats have also been found to be affected due to sex and age [29]. More detailed information concerning myostatin and adiponectin levels related to the effect of body weight would have been obtained through examining the sexes separately; however, sex was included as an independent variable in the multivariate analysis in this study. Second, we could not use nutrition data or the Food Frequency Questionnaire, because relevant data were frequently missing and could not be validated. Nutrition data are important for weight loss, and various studies have reported the relationship between myostatin plasma levels and food intake [30, 31]. One study reported no significant diet-related differences between myostatin plasma levels and insulin sensitivity in rats [30]. However, increased myostatin expression was observed in muscle following a high-fat diet intake in high-fat diet-induced obesity-susceptible mice, whereas myostatin expression levels decreased initially in muscle in high-fat diet-fed resistant mice [31]. Adiponectin exerts regulatory effects on GLU and lipid metabolism, insulin sensitivity, and inflammation, and plays an important role in the central regulation of appetite and eating behavior [32]. Food intake investigation might be important to demonstrate the relationship between myostatin and adiponectin levels and weight loss. Third, myostatin and adiponectin secretion levels in skeletal muscle and adipose tissue were not clear because these levels were measured using blood samples. However, blood sample evaluation is considered useful for clinical application given skeletal muscle and adipose tissue biopsy is invasive for patients. Finally, there were significant between-group differences in terms of patients’ medical histories concerning DM and hypertension at baseline. It has been reported that lower serum myostatin levels are independently associated with metabolic syndrome, obesity, dyslipidemia, and DM [33]. Future prospective studies should be considered involving patients with similar medical histories.

## Conclusion

This study showed that changes in serum myostatin levels were associated with changes in the rate of LBM and adiponectin levels. Additionally, changes in adiponectin levels independently predicted changes in myostatin levels. Therefore, myostatin and adiponectin might cross-talk and regulate changes in skeletal muscle and fat mass. These findings indicate that evaluating serum myostatin and adiponectin levels in clinical practice might be informative in predicting the effects of weight loss and in preventing the loss of skeletal muscle mass.

## Data Availability

The data that support the findings of this study are available on request from the corresponding author.

## Abbreviations

AT: Anaerobic threshold
BMI: Body mass index
GLU: Glucose
HbA1c: Glycosylated hemoglobin
HOMA-IR: Homeostasis model assessment of insulin resistance
IRI: Immunoreactive insulin
LBM: Lean body mass
MET: Metabolic equivalent
SFA: Subcutaneous fat area
VCO_2_: Carbon dioxide excretion volume
VFA: Visceral fat area
VO_2_: Oxygen uptake
